# Outpatient Antibiotic Prescribing Patterns and Appropriateness for Children in Primary Healthcare Settings in Beijing City, China, 2017–2019

**DOI:** 10.3390/antibiotics10101248

**Published:** 2021-10-14

**Authors:** Haishaerjiang Wushouer, Kexin Du, Shicai Chen, Yue Zhou, Bo Zheng, Xiaodong Guan, Luwen Shi

**Affiliations:** 1 Department of Pharmacy Administration and Clinical Pharmacy, School of Pharmaceutical Sciences, Peking University, Beijing 100191, China; kaiser@bjmu.edu.cn (H.W.); lavenderdkx@126.com (K.D.); zhouyuezhy@pku.edu.cn (Y.Z.); shilu@bjmu.edu.cn (L.S.); 2 International Research Center for Medicinal Administration (IRCMA), Peking University, Beijing 100191, China; 3 Department of Pharmacy, Luhe Teaching Hospital of Capital Medical University, Beijing 101100, China; chen6932@163.com; 4 Institute of Clinical Pharmacology, Peking University First Hospital, Beijing 100034, China; doctorzhengbo@163.com

**Keywords:** children, antibiotic use, appropriateness, primary health institutions

## Abstract

(1) Background: Few studies have focused on antibiotic use and appropriateness in children in primary health institutions (PHIs). This study aimed to identify the patterns and appropriateness of antibiotic use for children in PHIs in Beijing, China. (2) Methods: Outpatient prescriptions of 327 PHIs from 2017 to 2019 for patients <18 years old were collected. Prescriptions were described using quantity indicators. Antibiotics were categorized according to ATC classification J01 and Access, Watch, Reserve grouping. Appropriateness was reviewed by experts using three subtypes of irrational prescriptions (irregular, inappropriate, and abnormal). (3) Results: 20,618 prescriptions were collected in total. The antibiotic prescription rate (APR) was 15.1% (*N* = 3113). Among antibiotic prescriptions, J01FA Macrolides were the most used (*N* = 1068, 34.9%). The Watch group constituted 89.0% (*N* = 2818) of total antibiotic use. Bronchitis (*N* = 1059, 35.2%) was the most common diagnosis. A total of 292 instances of irrational antibiotic use were identified, with inappropriate prescriptions being the most prevalent subtype (*N* = 233, 79.8%). (4) Conclusion: Although APR for children in PHIs in Beijing was relatively low, the pattern of antibiotic use differed from other countries. Further studies are needed to optimize antibiotic use for children in PHIs under different levels of economic development.

## 1. Introduction

The selection pressure of antibiotics, driven by inappropriate antibiotic prescribing, generates antimicrobial resistance [1,2]. The development of antimicrobial resistance (AMR) is further accelerated by inappropriate antibiotic use and has become a growing public health threat worldwide, particularly for children [3,4]. An estimate from the China Antimicrobial Resistance Surveillance System (CARSS) showed that the proportions of third-generation cephalosporin-resistant *Escherichia coli* in China (CTX/CRO-R ECO) isolated from children and newborns in 2019 were 38.8% and 10.1%, respectively, which were higher than those from adults (28.8%) [5]. Appropriate antibiotic use in children is also critical, as there are limited antibiotic formulations suitable for this population.

China is among the largest consumers of antibiotics worldwide and has taken many measures to strengthen antimicrobials management [6,7,8,9]. However, most policies were implemented in secondary and tertiary hospitals, while primary healthcare institutions (PHIs) were subject to more localized management measures [10]. Since PHIs were not included in the national surveillance networks for both antibiotic use and AMR, understanding and addressing antibiotics use in PHIs can enhance the management of antibiotics use and AMR surveillance.

Beijing is the capital city of China and enjoys a high level of health system management. The Beijing Municipal Health Commission has made great efforts to promote rational antibiotic use in PHIs, including establishing a Prescription Review and Feedback (PRF) system. The PRF, one of the interventions to promote rational antibiotic use, was first introduced in PHIs in Beijing in 2014 [11]. Studies have shown that this approach is effective in controlling irrational antibiotic use in various settings across the world, regardless of their levels of economic development [12,13,14,15,16]. However, we found little evidence about the patterns and appropriateness of antibiotic use in children in PHIs [17,18]. To explore how antibiotics were used for children in PHIs, this study aims to determine the patterns and appropriateness of antibiotic prescriptions among children in PHIs in Beijing between 2017 and 2019.

## 2. Results

### 2.1. Selected Indicators of the Prescriptions

The selected indicators of all the sample prescriptions are described in Table 1. Of all the prescriptions (*N* = 1,809,616) extracted from 327 reporting PHIs of the Beijing Prescription Reviewing System of Community Healthcare Institutions (BPRSCHI) included in the study, a total of 20,618 prescriptions (1.6%) for children were extracted during the study period, among which 3113 prescriptions (15.1%) contained antibiotics. Among all antibiotic prescriptions, antibiotic combination prescriptions and prescriptions with antibiotic injections accounted for 1.6% (*N* = 50) and 8.6% (*N* = 269), respectively. Antibiotic prescriptions that were reviewed and rated as irrational accounted for 9.2% (*N* = 286) of all antibiotic prescriptions. The antibiotic prescription rate (APR) for children demonstrated a downward trend from 17.9% (*N* = 995) in 2017 to 13.9% (*N* = 1447) in 2019. The irrational antibiotic prescription rate showed a more substantial decrease from 14.0% (*N* = 139) to 4.8% (*N* = 69) during the study period.

### 2.2. Patterns of Antibiotic Use among Children

**Figure 1:**
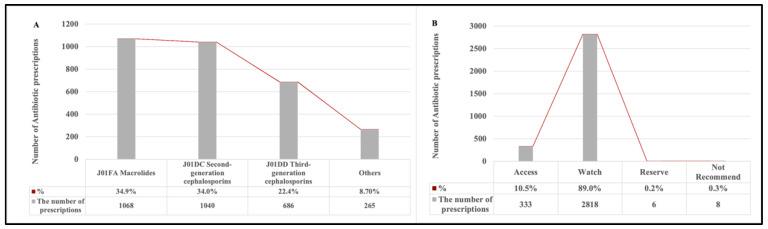
Patterns of antibiotic use among children, as described in the Anatomical Therapeutic and Chemical classification and the 2020 WHO EMLc Access, Watch, Reserve (AWaRe) grouping. (**A**).Patterns of antibiotic use among children described in the Anatomical Therapeutic and Chemical classification. (**B**). Patterns of antibiotic use among children described in the 2019 WHO EMLc Access, Watch, Reserve (AWaRe) grouping.

**Figure 2:**
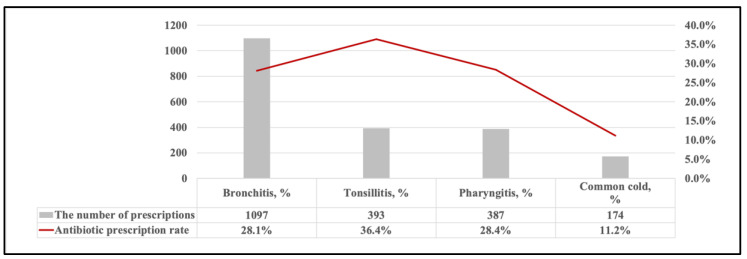
Antibiotic prescription rate of common infections in outpatient antibiotic prescriptions for children.

Among all antibiotic prescriptions for children, J01FA Macrolides was the most commonly prescribed antibiotics (*N* = 1068, 34.9%), followed by J01DC second-generation cephalosporins (*N* = 1040, 34.0%), and J01DD third-generation cephalosporins (*N* = 686, 22.4%) (Figure 1A). When using the 2020 WHO EMLc Access, Watch, Reserve (AWaRe) grouping to analyze the patterns of antibiotic use, prescriptions of the Watch group of antibiotics constituted most of the antibiotic prescriptions (*N* = 2818, 89.0%), followed by the Access group of antibiotics (*N* = 333, 10.5%). The Reserve group (*N* = 6, 0.2%) and Not Recommend group of antibiotics (*N* = 8, 0.3%) were barely prescribed (Figure 1B). Tonsillitis was the most frequent diagnosis shown on antibiotic prescriptions (*N* = 393, 36.4%), followed by pharyngitis (*N* = 387, 28.4%), bronchitis (*N* = 1097, 28.1%), and common cold (*N* =174, 11.2%). Bronchitis (*N* = 1059, 35.2%) was the most common diagnosis among commonly seen infectious conditions, as shown in Figure 2.

### 2.3. Appropriateness of Antibiotic Use for Children

As demonstrated in Table 2, among all antibiotic prescriptions, 9.2% (*N* = 286) were determined as irrational, amounting to a total of 292 incidences of inappropriate antibiotic use. When divided by types (see Table 2 for detailed definitions), inappropriate prescriptions accounted for 79.8% of all irrational prescriptions (*N* = 233), followed by irregular prescriptions (*N* = 59, 20.2%), and no prescriptions were reviewed as abnormal. Among the subtypes of inappropriate prescriptions, prescriptions with inappropriate usage and dosage (*N* = 145, 49.7%), inappropriate indication (*N* = 25, 8.6%), and inappropriate route of administration (*N* = 22, 7.5%) were most commonly mentioned. Among the subtypes of irregular prescriptions, non-conformity with the National Regulations on the Clinical Application of Antibiotics (*N* = 15, 5.1%), prescribing without a clinical diagnosis or with an incomplete clinical diagnosis (*N* = 13, 4.5%), and unspecified date of birth for infants and newborns (*N* = 8, 2.7%) ranked as the top three.

## 3. Discussion

In this cross-sectional observational study, we identified the patterns of antibiotic use and evaluated the appropriateness of antibiotic prescriptions of PHIs in Beijing, China. We found that the pediatric antibiotic prescribing rate in PHIs in Beijing (15.1%) was lower than that in France (26.1%) [19], Australia (23%) [20], the Netherlands (29%) [21], Germany (38.6%) [22], and the United Kingdom (36.2%) [23], but was slightly higher than in Italy (8.81%) [24]. The GDP per capita of Beijing was 10,484 U.S. dollars, roughly equivalent to that of Malaysia (10,270 U.S. dollars) and Russia (10,037 U.S. dollars) [25]. This relatively low antibiotic prescription rate for children in community settings in Beijing could be attributable to the better healthcare management capacity of PHIs compared with the countries above. It could also be attributable to China’s measures to control antibiotic use, including establishing restrictions on antibiotic allocation to health facilities [26], public reporting of inappropriate antibiotic use [27], and providing continuous professional training for health professionals [28]. A study showed that the outpatient APR was 35.5% in PHIs in Beijing during 2009–2011, with only 42.4% of the antibiotic prescriptions considered as appropriate [29]. After the implementation of the PRF process in 2014, the APR in children in Beijing showed a sharp decrease by 15.1% in our study, suggesting the effectiveness of PRF in promoting appropriate antibiotic use among children seen in PHIs. However, as previously mentioned, metropolitan cities such as Beijing and Shenzhen are privileged to have PHIs with better management capacity, which pave the way for PRF implementation and thus control of inappropriate antibiotic use [30]. In less-resourced settings such as the rural areas in Guangxi province, PHIs have a more limited management capacity but are confronted with similar, if not greater, challenges from inappropriate antibiotic use. In rural China, APR was over 30% for children diagnosed with upper respiratory infections, suggesting a high rate of inappropriate antibiotic use among children [31]. PRF has the potential to lower regional disparities in controlling inappropriate antibiotic use, especially in PHIs, as qualitative research suggested that residents in rural areas were more likely to seek care and continue treatment in PHIs compared with their urban counterparts [32]. Still, PRF implementation is context-specific and needs to be contextualized to local needs and capacity. More studies are therefore needed to verify the effectiveness of PRF in different settings.

J01FA Macrolides were the most prescribed antibiotics in our study sample. This finding illustrated a difference in antibiotic utilization patterns between communities in Beijing and in other developed countries. In Europe, the most consumed antibiotics in community settings in 2019 were penicillins across all countries but Slovakia, with the proportion ranging from 27% (Slovakia) to 66% (Denmark) of the total antibiotic consumption [33]. In Beijing, we found that cephalosporins and other beta-lactams (ATC group J01D) accounted for 56.4% of the total pediatric antibiotic use, nearly twice as high as in Europe (Slovakia, 27%). Moreover, 34.9% of prescribed antibiotics in Beijing were lincosamides and streptogramins, which is far more than that in Europe (ATC group J01F, 26%), according to sales and reimbursement data. These discrepancies in antibiotic utilization reflect differences in antibiotic selection among primary pediatric caregivers from different countries due to factors such as patient pressure, time constraints, diagnosis uncertainty, and so forth [34]. Moreover, the abundant use of macrolides and the second- and third-generation cephalosporins could be attributed to the following factors: first, in China, prescribing penicillin requires a skin test. The inconvenience of using penicillins due to over-estimation of penicillin allergy rates could impel some clinicians to replace penicillins with cephalosporins or other broad-spectrum antibiotics [35,36]. Second, azithromycin is more child-friendly in terms of form for convenient use. Third, due to the shortage of guidelines related to respiratory self-limiting disease for the pediatric population, as well as vague expression in guidance for drug choice in the existing ones, physicians can barely obtain practical information from guidelines. These factors may result in the free choice of antibiotics under pressures such as patient demands [34]. However, cephalosporins are not recommended as the first-line treatment for acute respiratory infections (ARI) in children in Chinese guidelines. This prescribing manner suggested a low guideline adherence in physicians in PHIs, whose prescribing capacity would need further training processes and other interventions to improve.

Despite the relatively low APR for children compared with other countries and the decreasing rate of inappropriate antibiotic use, our results suggest that control measures of antibiotic use await further improvements. Our results showed that 28.1% of the prescriptions for bronchitis and 11.2% of the prescriptions for the common cold contained antibiotics. Since antibiotics are not indicated in these two syndromes, and the sample prescriptions were collected based on a reporting mechanism, the inappropriateness may be underestimated. Besides, the Watch group antibiotics were the most prescribed antibiotics for children in our study. There is still a large gap in the utilization of the Access group antibiotics, which is recommended by the WHO to account for at least 60% of total antibiotic use by 2023 [37]. Another possible contributor to this large gap in antibiotic use compared with the WHO recommendation might be the differences between the Chinese formulation restriction policy and the AWaRe classification [26]. Moreover, skewness was also shown in the distribution of the appropriateness reviewing process results. The subtype of inappropriate prescription (79.8%) was the main error, most of which was attributable to inappropriate usage and dosage (49.7%). The lack of pediatric-specific comparative data, uncertainty in pediatric dosing regimens for several agents, and a relative lack of new antibiotics with pediatric indications collectively present unique challenges for antibiotic use in children [38]. There is an urgent need to develop new antibiotics and dosage regimens for children and to enhance their accessibility, as they are essential to child health across the world, not only in China.

Our study has several limitations. First, the information of medical history, secondary diagnosis, and pathogenic examination could not be retrieved by the reviewers from the BPRSCHI database, which might introduce bias to the results in the reviewing process. Second, because the prescription-reviewing process was conducted based on prescription per visit instead of per patient, the linkage between prescriptions and patients was not accessible. Nonetheless, the analysis based on prescriptions was accurate enough to reflect the patterns in antibiotic use for children in PHIs. Third, although most of the community healthcare centers (CHCs) were included in the BPRSCHI database, the selection of PHIs could introduce selection bias due to the imbalanced distribution of CHCs and community healthcare stations (CHSs) in the sample PHIs. Fourth, the study sample may not be fully representative of the general child population in Beijing as parents might prefer to seek care at higher-level medical institutions instead of PHIs, potentially leading to overestimated results in this study. Fifth, socioeconomic information on the doctors, which could affect prescribing behaviors [34], was not included due to data accessibility, which may introduce bias to the results. Sixth, although the sampling process was based on a systematic randomized methodology, we could not avoid potential selection biases in the reporting process. However, the random selection process could maximize the representativeness of the prescriptions. Seventh, due to the absence of ICD coding, we could not present all the diagnoses of the prescriptions. Nonetheless, prescriptions of the four commonly seen diagnoses were the majority of the prescriptions (65.9%, *N* = 2057).

## 4. Materials and Methods

### 4.1. Methods

#### 4.1.1. Study Design

We conducted a cross-sectional observational study to quantify the pattern and appropriateness of antibiotic prescriptions for children in 327 PHIs in Beijing from January 2017 to December 2019.

#### 4.1.2. Study Setting and Data Source

As the capital of China, Beijing had 21.5 million residents (12.6% of whom were aged under 19) and 2075 PHIs (including 345 CHCs and 1730 CHSs) with 68.3 million visits in 2019 (25.8% of total hospital visits). PHIs are designed to be the first level of contact of patients with the national health system. All PHIs in Beijing are outpatient clinics with very little inpatient capacity (only 26,000 patients were discharged from PHIs in 2019), providing basic outpatient clinical care and public health services to individuals and families residing in the community.

All reported prescriptions from the sample PHIs (16.0% of total PHIs) were extracted from the Beijing Prescription Reviewing System of Community Healthcare Institutions (BPRSCHI) database. BPRSCHI was established by Beijing Health Commission in 2014 for routine online prescription reviews of the sample prescriptions that are reported to the database. The selection of PHIs in BPRSCHI is based on a convenience sampling method while considering the geographical representativeness of the city. The BPRSCHI database covered 327 PHIs; most of them were community health centers (321 out of 345) during 2017–2019. A sampling software is embedded in the information system of all PHIs. A total of 100 prescriptions were randomly selected from each PHI monthly using a systematic sampling method, with the sampling interval calculated by dividing the number of total prescriptions by 100.

#### 4.1.3. Data Collection

All prescription data in BPRSCHI (*N* = 1,809,616) from January 2017 to December 2019 were extracted. We selected the prescriptions for children for further analysis. We derived data on prescription code, area text, prescribing date, age, gender, medical insurance status, diagnoses, medication, specification, dosage, administration route, and the result of the reviewing process given by the BPRSCHI review team. Electronic prescriptions were digitally transferred from the database and were double-checked by our researchers. The selection of the antibiotic prescriptions for children is shown in Figure S1.

In this study, children were defined as patients under 18 years old. A prescription was defined as all the medicines prescribed to a patient during one visit. Antibiotic prescriptions were defined as the prescriptions that contained at least one antibiotic according to Anatomical Therapeutic and Chemical (ATC) classification J01 [37]. Due to the absence of standardization of diagnosis, namely the adoption of International Classification of Diseases (ICD) coding in PHIs, it is difficult to standardize all the diagnoses of the prescriptions. Therefore, we collected data on four commonly seen infectious conditions by searching the following keywords in diagnoses: “*Qiguanyan*”, “*Biantaotiyan*”, “*Yanyan*”, “*Ganmao*” (meaning bronchitis, tonsillitis, pharyngitis, and common cold, respectively).

### 4.2. Measurements

#### 4.2.1. Indicators

The prescriptions for children were described using the following indicators. The percentage of antibiotic-containing prescriptions was calculated by dividing the number of prescriptions that contained at least one antibiotic by the total number of prescriptions. The percentage of antibiotic combination prescriptions was calculated by dividing the number of prescriptions with more than one antibiotic by the total number of antibiotic prescriptions. The percentage of prescriptions with antibiotic injections was calculated by dividing the number of prescriptions that contained at least one antibiotic injection by the total number of antibiotic prescriptions. The percentage of irrational antibiotic prescriptions was calculated by dividing the number of prescriptions reviewed as irrational by the total number of antibiotic prescriptions.

#### 4.2.2. Patterns of Antibiotic Use Indicated by the Sample Prescriptions

We described the patterns of antibiotic use by calculating the proportion of the antibiotic prescriptions, classified according to Anatomical Therapeutic and Chemical (ATC) classification J01 [39]. We also adopted the 2019 WHO EMLc Access, Watch, Reserve (AWaRe) grouping to analyze the proportion of antibiotic use by AWaRe categories [40]. We further calculated the antibiotic prescription rate (APR) for the selected diagnoses by dividing the number of antibiotic prescriptions for the given diagnosis by the number of prescriptions for the same diagnosis.

#### 4.2.3. Appropriateness of the Antibiotic Prescriptions

We analyzed the appropriateness of the antibiotic prescriptions by the result of the reviewing process. According to “Regulation Standard for Hospital Prescription Review” issued by the Chinese Ministry of Health in 2010 [11], the PRF process is conducted manually on a monthly basis by a review team consisting of multidisciplinary healthcare professionals, including physicians, pharmacists, and microbiologists, as well as experts of medical management from tertiary hospitals. Following the “Guideline for the prescription review process of Beijing healthcare institutions”, the review is conducted based on the clinical pathways, medication, and clinical treatment guidelines, as well as the medication manufactory instructions. As quality-control measures, the Hospital Medication and Therapeutics Committee is responsible for providing training to the review team before conducting the review, and Medical Quality Committee is responsible for quality evaluation after the reviewing process periodically.

In light of the “Regulation Standard for Hospital Prescription Review”, an irrational prescription is a prescription with inferior quality in writing or with inappropriate use of medications (including indication, selection of drugs, administration route, usage and dosage, drug–drug interaction, and incompatibility of drugs) compared with relevant law and technical specifications. An irrational prescription could be categorized as one of the following subtypes based on whether the use of antibiotics adheres to standards: irregular prescription, inappropriate prescription, and abnormal prescription based on different types of mistakes or inappropriateness of the prescription. Irregular prescriptions refer to prescriptions with unclear, non-standard, ambiguous, or missing components of a standard prescription. Inappropriate prescriptions refer to prescriptions with unreasonable conditions in drug selection, indications, usage and dosage, drug interaction, etc. Abnormal prescriptions refer to the choice of drug use beyond the general principles of drug therapy or the lack of justification of prescriptions. The detailed criteria of these three subtypes of irrational prescriptions are shown in Table S1.

#### 4.2.4. Data Analysis

Descriptive statistics were reported throughout. Statistical analysis was performed using STATA V.15.1 (StataCorp, TX, USA) and Excel 2016.

#### 4.2.5. Ethics Approval

Ethics committee approval was obtained from Peking University Institution Review Board (IRB00001052-21048). Patient consent was waived because no contact with patients was conducted and patient anonymity was assured.

## 5. Conclusions

This study described the patterns in antibiotic use among children in PHIs in Beijing. Although a relatively low level of antibiotic utilization was found, the fact of an extremely high proportion of Watch group prescriptions raises concerns of potential inappropriateness. Besides that, the patterns in PHIs in Beijing differ significantly from settings with comparable levels of economic development. To combat drug resistance and irrational use of antibiotics in children, more research should focus on the use of antibiotics in children in primary care settings and its influencing factors in areas with different economic levels in order to optimize the use of antibiotics in children at the primary level and curb the occurrence of drug resistance in children.

## Figures and Tables

**Table 1:** Selected indicators of prescriptions for children from all PHIs covered by Beijing Prescription Reviewing System of Community Healthcare institutions, 2017–2019.

Indicators	2017	2018	2019	Total
Prescriptions for children, n	5569	4627	10,422	20,618
Antibiotic-containing prescriptions, n	995	671	1447	3113
(% of prescriptions)	(17.9%)	(14.5%)	(13.9%)	(15.1%)
Prescriptions with antibiotic combinations, n	18	12	20	50
(% of antibiotic prescriptions)	(1.8%)	(1.8%)	(1.4%)	(1.6%)
Prescriptions with antibiotic injections, n	78	50	141	269
(% of antibiotic prescriptions)	(7.8%)	(7.5%)	(9.7%)	(8.6%)
Irrational antibiotic prescriptions, n	139	78	69	286
(% of antibiotic prescriptions)	(14.0%)	(11.6%)	(4.8%)	(9.2%)

**Table 2:** Frequency of three types of irrational prescription of sample antibiotic prescriptions for children, 2017–2019.

No.	Irrational Prescription Type	Frequency *N* = 292	Proportion of 100.0%
1	Irregular prescription	*N* = 59	20.2
1-1	Missing elements, non-standardized, or illegible writing.	4	1.4
1-3	Absence of a prescription suitability review	1	0.3
1-4	Unspecified date of birth for infants and newborns (in months or days)	8	2.7
1-7	Illegible writing of dosage, specifications, usage, unit in the prescription	6	2.1
1-8	Ambiguous expression concerning dosage and/or use (e.g., “follow the doctor’s advice”, “self-medicated”, etc.)	1	0.3
1-10	Prescribing without a clinical diagnosis or with an incomplete clinical diagnosis	13	4.5
1-14	Non-conformity with the National Regulations on the Clinical Application of Antibiotics	15	5.1
2	Inappropriate prescription	*N* = 233	79.8
2-1	Inappropriate indication	25	8.6
2-2	Inappropriate selection of drugs	19	6.5
2-3	Inappropriate route of administration	22	7.5
2-5	Inappropriate usage and dosage	145	49.7
2-6	Inappropriate combined use of drugs	9	3.1
2-7	Repeated administration	6	2.1
2-8	Incompatibility or adverse interaction	4	1.4
2-9	Other inappropriate situations	3	1.0

Note: The subgroup of irrational use was defined by the “Regulation Standard for Hospital Prescription Review” 2010.

## Data Availability

The data that support the findings of this study are available from the Beijing community hospital prescription review workgroup, but restrictions apply to the availability of these data, which were used under license for the current study, and so are not publicly available. Data are, however, available from the authors upon reasonable request and with permission of the Beijing community hospital prescription review workgroup.

## Supplementary Materials

The following are available online at https://www.mdpi.com/article/10.3390/antibiotics10101248/s1, Figure S1: The selection of antibiotic prescriptions. Table S1: Definition of the three types of irrational prescriptions.

## References

[1] Wushouer H., Zhou Y., Zhang X., Fu M., Fan D., Shi L., Guan X. (2020). Secular trend analysis of antibiotic utilisation in China’s hospitals 2011–2018, a retrospective analysis of procurement data. Antimicrob. Resist. Infect. Control.

[2] Angebault C., Andremont A. (2013). Antimicrobial agent exposure and the emergence and spread of resistant microorganisms: Issues associated with study design. Eur. J. Clin. Microbiol. Infect. Dis..

[3] Zhao H., Wei L., Li H., Zhang M., Cao B., Bian J., Zhan S. (2021). Appropriateness of antibiotic prescriptions in ambulatory care in China: A nationwide descriptive database study. Lancet Infect. Dis..

[4] Laxminarayan R., Amabile-Cuevas C.F., Cars O., Evans T., Heymann D.L., Hoffman S., Holmes A., Mendelson M., Sridhar D., Woolhouse M. (2016). UN High-Level Meeting on antimicrobials—What do we need?. Lancet.

[5] China Antimicrobial Resistance Surveillance System (2021). Epidemiological change in carbapenem-resistant *Klebsiella pneumoniae*: Surveillance report from China Antimicrobial Resistance Surveillance in 2014–2019. Chin. J. Infect. Control.

[6] (2019). National Guiding Principles for Antimicrobial. http://www.gov.cn/xinwen/2015-08/27/content_2920799.htm.

[7] Ministry of Health of China National Guiding Principles for Antimicrobial. http://www.sda.gov.cn/WS01/CL0055/10126.html.

[8] Ministry of Health of China Notification on Establishing Surveillance Network for Antibiotic Use and Antimicrobial Resistance. http://www.nhfpc.gov.cn/zwgkzt/wsbysj/200804/18487.shtml.

[9] Ministry of Health of China Notification on Releasing Clinical Guidance of National Essential Medicine and National Essential Medicine Formulary. http://www.nhfpc.gov.cn/zwgkzt/wsbysj/201005/47420.shtml.

[10] Xiao Y. (2018). Antimicrobial Stewardship in China: Systems, Actions and Future Strategies. Clin. Infect. Dis..

[11] National Health Commission of People’s Republic of China Regulation Standard for Hospital Prescription Review. http://www.nhc.gov.cn/wjw/ywfw/201306/094ebc83dddc47b5a4a63ebde7224615.shtml.

[12] Friedman N.D. (2013). Antimicrobial Stewardship: The Need to Cover All Bases. Antibiotics.

[13] Cosgrove S.E., Seo S.K., Bolon M.K., Sepkowitz K.A., Climo M.W., Diekema D.J., Speck K., Gunaseelan V., Noskin G.A., Herwaldt L.A. (2012). Evaluation of postprescription review and feedback as a method of promoting rational antimicrobial use: A multicenter intervention. Infect. Control Hosp. Epidemiol..

[14] Takamatsu A., Yao K., Murakami S., Tagashira Y., Hasegawa S., Honda H. (2020). Barriers to Adherence to Antimicrobial Stewardship Postprescription Review and Feedback For Broad-Spectrum Antimicrobial Agents: A Nested Case-Control Study. Open Forum Infect. Dis..

[15] Tamma P.D., Avdic E., Keenan J.F., Zhao Y., Anand G., Cooper J., Dezube R., Hsu S., Cosgrove S.E. (2017). What Is the More Effective Antibiotic Stewardship Intervention: Preprescription Authorization or Postprescription Review With Feedback?. Clin. Infect. Dis..

[16] Zhou J., Ma X. (2019). A survey on antimicrobial stewardship in 116 tertiary hospitals in China. Clin. Microbiol. Infect..

[17] Lee K.R., Bagga B., Arnold S.R. (2016). Reduction of Broad-Spectrum Antimicrobial Use in a Tertiary Children’s Hospital Post Antimicrobial Stewardship Program Guideline Implementation. Pediatric Crit. Care Med..

[18] Velasco-Arnaiz E., Simó-Nebot S., Ríos-Barnés M., López Ramos M.G., Monsonís M., Urrea-Ayala M., Jordan I., Mas-Comas A., Casadevall-Llandrich R., Ormazábal-Kirchner D. (2020). Benefits of a Pediatric Antimicrobial Stewardship Program in Antimicrobial Use and Quality of Prescriptions in a Referral Children’s Hospital. J. Pediatrics.

[19] Trinh N.T.H., Cohen R., Lemaitre M., Chahwakilian P., Coulthard G., Bruckner T.A., Milic D., Levy C., Chalumeau M., Cohen J.F. (2020). Community antibiotic prescribing for children in France from 2015 to 2017: A cross-sectional national study. J. Antimicrob. Chemother..

[20] Howarth T., Brunette R., Davies T., Andrews R.M., Patel B.K., Tong S., Barzi F., Kearns T.M. (2020). Antibiotic use for Australian Aboriginal children in three remote Northern Territory communities. PLoS ONE.

[21] van Aerde K.J., de Haan L., van Leur M., Gerrits G.P., Schers H., Moll H.A., Hagedoorn N.N., Herberg J.A., Levin M., Rivero-Calle I. (2021). PERFORM Consortium. Respiratory Tract Infection Management and Antibiotic Prescription in Children: A Unique Study Comparing Three Levels of Healthcare in the Netherlands. Pediatric Infect. Dis. J..

[22] Dik J.W., Sinha B., Friedrich A.W., Lo-Ten-Foe J.R., Hendrix R., Köck R., Bijker B., Postma M.J., Freitag M.H., Glaeske G. (2016). Cross-border comparison of antibiotic prescriptions among children and adolescents between the north of the Netherlands and the north-west of Germany. Antimicrob. Resist. Infect. Control.

[23] de Bie S., Kaguelidou F., Verhamme K.M., De Ridder M., Picelli G., Straus S.M., Giaquinto C., Stricker B.H., Bielicki J., Sharland M. (2016). Using Prescription Patterns in Primary Care to Derive New Quality Indicators for Childhood Community Antibiotic Prescribing. Pediatric Infect. Dis. J..

[24] Di Mario S., Gagliotti C., Buttazzi R., Cisbani L., Di Girolamo C., Brambilla A., Moro M.L. (2018). regional working group “Progetto ProBA-Progetto Bambini e Antibiotici-2014”. Observational pre-post study showed that a quality improvement project reduced paediatric antibiotic prescribing rates in primary care. Acta Paediatr..

[25] International Monetary Fund World Economic Outlook Database. https://www.imf.org/en/Publications/WEO/weo-database/2021/April/download-entire-database.

[26] Xiao Y. (2012). Antibiotic formulary restriction: Theory & practice. Beijing Med. J..

[27] Tang Y., Liu C., Zhang X. (2016). Public reporting as a prescriptions quality improvement measure in primary care settings in China: Variations in effects associated with diagnoses. Sci. Rep..

[28] Wei X., Zhang Z., John D.W., Joseph P.H., Zeng J., Deng S., Zhou Y., Yin J., James N.N., Sun Q. (2017). Effect of a training and educational intervention for physicians and caregivers on antibiotic prescribing for upper respiratory tract infections in children at primary care facilities in rural China: A cluster-randomised controlled trial. Lancet Glob. Health.

[29] Wang J., Wang P., Wang X., Zheng Y., Xiao Y. (2014). Use and Prescription of Antibiotics in Primary Health Care Settings in China. JAMA Intern. Med..

[30] Gong Y., Li H., Yang H., Tan K., Liu W., Li X., Wu J., Zhang G., Yin X. (2021). Evaluation of the Quality of Antibiotic Prescribing in Primary Care: A Multicenter Longitudinal Study From Shenzhen, China. Front. Pharmacol..

[31] Zhang Z., Hu Y., Zou G., Lin M., Zeng J., Deng S., Zachariah R., Walley J., Tucker J.D., Wei X. (2017). Antibiotic prescribing for upper respiratory infections among children in rural China: A cross-sectional study of outpatient prescriptions. Glob. Health Action.

[32] Liu Y., Zhong L., Yuan S., van de Klundert J. (2018). Why patients prefer high-level healthcare facilities: A qualitative study using focus groups in rural and urban China. BMJ Glob. Health.

[33] European Centre for Disease Prevention and Control (2020). Antimicrobial Consumption in the EU/EEA—Annual Epidemiological Report 2019.

[34] Zetts R.M., Stoesz A., Smith B.A., Hyun D.Y. (2018). Out patient Antibiotic Use and the Need for Increased Antibiotic Stewardship Efforts. Pediatrics.

[35] Joint Task Force on Practice Parameters (2010). Drug allergy: An updated practice parameter. Ann. Allergy Asthma Immunol..

[36] Meng J., Wang L. (2016). Penicillin allergy and diagnostic methods. Chin. J. Allergy Clin. Immunol..

[37] World Health Organization (2017). WHO Model List of Essential Medicines (20th List). https://www.who.int/medicines/publications/essentialmedicines/20th_EML2017.pdf?%20ua=.

[38] Chiotos K., Hayes M., Gerber J.S., Tamma P.D. (2020). Treatment of Carbapenem-Resistant Enterobacteriaceae Infections in Children. J. Pediatric Infect. Dis. Soc..

[39] World Collaborating Centre for Drug Statistics Methodology Guidelines for ATC classification and DDD Assignment 2019. https://www.whocc.no/filearchive/publications/2019_guidelines_web.pdf.

[40] WHO 2019 AWaRe Classification Antibiotics [EB/OL]. https://www.who.int/medicines/news/2019/WHO_releases2019AWaRe_classification_antibiotics/en/.

