# Supplementary material for: Outpatient Antibiotic Prescribing Patterns and Appropriateness for Children in Primary Healthcare Settings in Beijing City, China, 2017–2019
Source: Antibiotics (Basel). 2021 Oct 14;10(10):1248. doi: 10.3390/antibiotics10101248 (PMC8532681; doi:10.3390/antibiotics10101248)
Supplement: Supplementary file 1 [file antibiotics-10-01248-s001.zip › Supplement 2- antibiotics -1364266.pdf]

Table S1. Definition of the three types of irrational prescriptions.

**Types of Irrational Prescriptions**	**Code**	**Definition**
Irregular prescriptions	1-1	Missing elements, non-standard or illegible writing.
	1-2	The physician's signature or seal did not meet the standard or is inconsistent with his/her previous signature or seal sample.
	1-3	Absence of a prescription suitability review
	1-4	Unspecified date of birth for infants and newborns (in months or days)
	1-5	Western medicines, Chinese patent medicines and Chinese herbal medicines are not prescribed separately.
	1-6	Prescribed without using the drug's regulatory name.
	1-7	Illegible writing of dosage, specifications, usage, unit in the prescription
	1-8	Ambiguous expression concerning dosage and/or use (e.g., "follow the doctor's advice", "self-medicated", etc.)
	1-9	Modification to the prescription is not signed and/or dated, or the reasons for the

		overdose are not indicated and/or re-signed.
	1-10	Prescribing without a clinical diagnosis or with an incomplete clinical diagnosis
	1-11	More than five medicines in a single outpatient and emergency prescription.
	1-12	Prescribing more than exceed 7 days of dosage for outpatient prescriptions or more than 3 days of dosage for emergency prescriptions; or extending prescriptions for patients with chronic diseases, elderly diseases or special circumstances without specifying reasons.
	1-13	Prescribing anesthetic drugs, psychotropic drugs, toxic drugs for medical use, radioactive drugs, and other drugs under special control without implementing the relevant provisions.
	1-14	Non-conformity with the *National Regulations on the Clinical Application of Antibiotics*
	1-15	Chinese traditional medicine prepared pieces are not arranged in the order of "king, minister, assistant, and envoy", or the special requirements of drug dispensing and decocting are not marked as required.

	1-16	Prescribing without Chinese medicine diagnosis, not including both disease name and category.
Inappropriate prescription	2-1	Inappropriate indications
	2-2	Inappropriate selection of drugs
	2-3	Inappropriate route of administration
	2-4	Not preferring national essential medicines without valid reasons
	2-5	Inappropriate usage and dosage
	2-6	Inappropriate combined use of drugs
	2-7	Repeated administration
	2-8	Contraindications or adverse interactions
	2-9	Other inappropriate situations
Abnormal prescription	3-1	No indication
	3-2	Prescribing high-priced drugs without valid reasons
	3-3	Off-label use without valid reasons
	3-4	Prescribing two or more drugs with the same pharmacological effect for the same patient without valid reasons
