# Supplementary figures and images for: Outpatient Antibiotic Prescribing Patterns and Appropriateness for Children in Primary Healthcare Settings in Beijing City, China, 2017–2019
Source: Antibiotics (Basel). 2021 Oct 14;10(10):1248. doi: 10.3390/antibiotics10101248 (PMC8532681; doi:10.3390/antibiotics10101248)

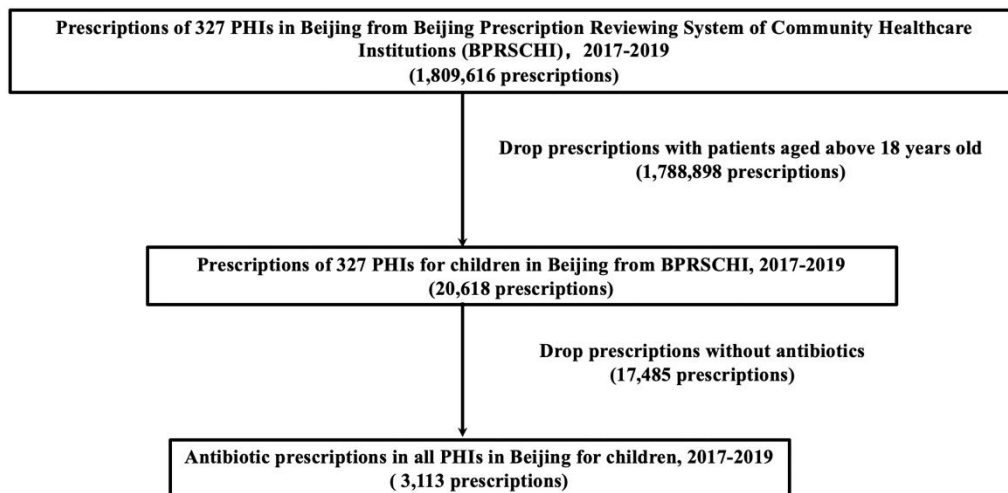

**Figure S1.** Selection of antibiotic prescriptions for children.

Supplement: Supplementary file 1 [file antibiotics-10-01248-s001.zip › Supplement 1- antibiotics-1364266.pdf]
